# Microbial communities and pharmaceutical contaminants in the water-soil–plant continuum of sugarcane crops irrigated with contaminated waters from the Cauca River Valley, Colombia

**DOI:** 10.1007/s11356-026-37722-y

**Published:** 2026-06-25

**Authors:** Juan Ceballos-Castillo, Rodrigo A. Echeverry-Gallego, Diana Martínez-Pachón, Alejandro Moncayo-Lasso, Javier Vanegas

**Affiliations:** Research Group in Biological and Chemical Sciences, Faculty of Sciences, Universidad Antonio Nariño, Cra 1 Este No. 47 a 15, Bogotá, DC Colombia

**Keywords:** Plant-associated microbiome, Emerging contaminants, Microbial transfer, Pharmaceutical residues, Irrigation water quality, Food security

## Abstract

“Agricultural irrigation with contaminated water poses a major risk to food security and public health. This study examined contaminant transfer across the water–soil–plant continuum in sugarcane crops irrigated with water from the Cauca River, Colombia. Microbial community composition was characterized using 16S rRNA gene sequencing, while pharmaceutical active compounds (PhACs) in irrigation water and sugarcane plants were quantified by UHPLC–MS/MS. Microbial analyses revealed a potential biological pathway of microorganisms from water and soil into plant endophytic tissues. Bacterial diversity decreased markedly, from more than 400 genera identified in water and soil to only 64 genera detected within endophytic tissues. Nine potentially pathogenic genera-including *Escherichia–Shigella*, *Pseudomonas*, and *Bacillus*- were found across all matrices, suggesting microbial internalization. Bioinformatic predictions indicated the presence of genes associated with virulence traits (e.g., antimicrobial resistance and biofilm formation) as well as beneficial functions such as nitrogen fixation and phosphate solubilization. In parallel, high concentrations of PhACs were detected in irrigation water, including valsartan (up to 1309 ng L⁻^1^) and diclofenac (up to 969 ng L⁻^1^), whereas none or only low concentration detected in sugarcane tissues. Overall, the primary risk to human health appears to stem not from chemical uptake but from microbial infiltration shaped by environmental pressures. Therefore, managing plant-associated microbiomes is essential to mitigate health risks while harnessing biotechnological potential for sustainable agriculture.”

**Supplementary Information:**

The online version contains supplementary material available at 10.1007/s11356-026-37722-y.

## Introduction

The intensification of sugarcane (*Saccharum officinarum*) cultivation in the Cauca Valley, the primary production hub in Colombia (De Matos et al. 2020; Babu et al. 2022), is intrinsically linked to the degradation of regional water resources. In particular, the widespread use of irrigation water from the Cauca River—a watershed that receives substantial municipal, industrial, and agricultural effluents with limited treatment (Hurtado & Vélez-Torres 2020)—creates a contamination continuum from water to soil and ultimately to the plant. This contamination pathway, driven by discharges from urban centers such as Cali and Jamundí, promotes the introduction of both chemical contaminants (e.g., agrochemicals and heavy metals) and biological agents into agricultural systems. As a result, irrigation acts as a direct vector of exposure, posing combined risks to food security and ecosystem health and underscoring the need for an integrated environmental and microbiological assessment (Cortés-Landázury & Gómez-Sánchez 2017).

In the agricultural context, the transfer of microorganisms via irrigation establishes a direct colonization route that extends from the water into the plant’s interior. Bacteria present in contaminated sources can establish themselves in the rhizosphere and become internalized as endophytes within plant tissues, including enteric pathogens such as *Salmonella*, which can persist despite washing procedures (Li et al. 2022; Kljujev et al. 2018). In sugarcane crops, the presence of opportunistic pathogens like *Burkholderia gladioli* and *B. cepacia* has been documented (Vanegas & Rodríguez 2018; Zheng et al. 2023). However, the composition and functionality of these invading microbial communities in the Cauca Valley, where water quality is a critical factor, remain a fundamental knowledge gap.

This biological risk cannot be understood in isolation, as it is intrinsically modulated by constant chemical pressure. The waters of the Cauca River carry a significant load of pharmaceutical active compounds (PhACs), herbicides, and heavy metals such as cadmium and mercury (Zapata Rivera et al. 2018; Enamorado-Montes et al. 2022). These xenobiotics not only pose a risk of bioaccumulation but also act as potent selective agents, capable of favoring microorganisms with antibiotic resistance mechanisms and metabolic capabilities for contaminant degradation (Mittal et al. 2019; Sodhi et al. 2020). This coexistence of biological and chemical contaminants can generate synergistic effects that amplify the overall risk, a complex phenomenon requiring integrated characterization through high-throughput molecular and analytical techniques.

Interestingly, the same selective pressure that drives health risks may also enrich the microbiome with valuable biotechnological potential. In contaminated environments, bacteria such as *Pseudomonas* spp. and *Bacillus* spp. often develop metabolic capabilities to degrade organic pollutants and, simultaneously, produce plant growth-promoting metabolites as a survival mechanism (Kumar & Chandra 2020; Leelastwattanagul et al. 2023; Echeverry-Gallego et al. 2024). Therefore, the microbial community of the Cauca Valley agroecosystem, far from being merely a reservoir of pathogens, could represent an endogenous source of resources for developing bioremediation and sustainable agriculture strategies.

A fundamental knowledge gap exists regarding how the microbiome is transferred and structured along the water-soil-plant continuum under the selective pressure of PhACs. This study hypothesizes that biological and chemical contaminants present in the Cauca River irrigation water are transferred to sugarcane soil and tissues, which could pose both health risks and biotechnological opportunities. To address this hypothesis, we employed a metagenomic approach based on 16S rRNA gene sequencing and the PhACs via ultra-high-performance liquid chromatography coupled to triple quadrupole mass spectrometry (UHPLC-MS/MS). The primary objective was to characterize the composition of bacterial communities in irrigation water, rhizospheric soil, and plant tissues, as well as to identify xenobiotics, in order to establish contaminant transfer routes and significant microbial interactions. Our study extends previous research focused on individual matrices by providing an integrated view of the entire system, contributing to the development of agricultural management strategies that minimize health and environmental risks associated with the use of contaminated water in sugarcane production systems.

## Materials and methods

### Study area and sampling

**Fig. 1:**
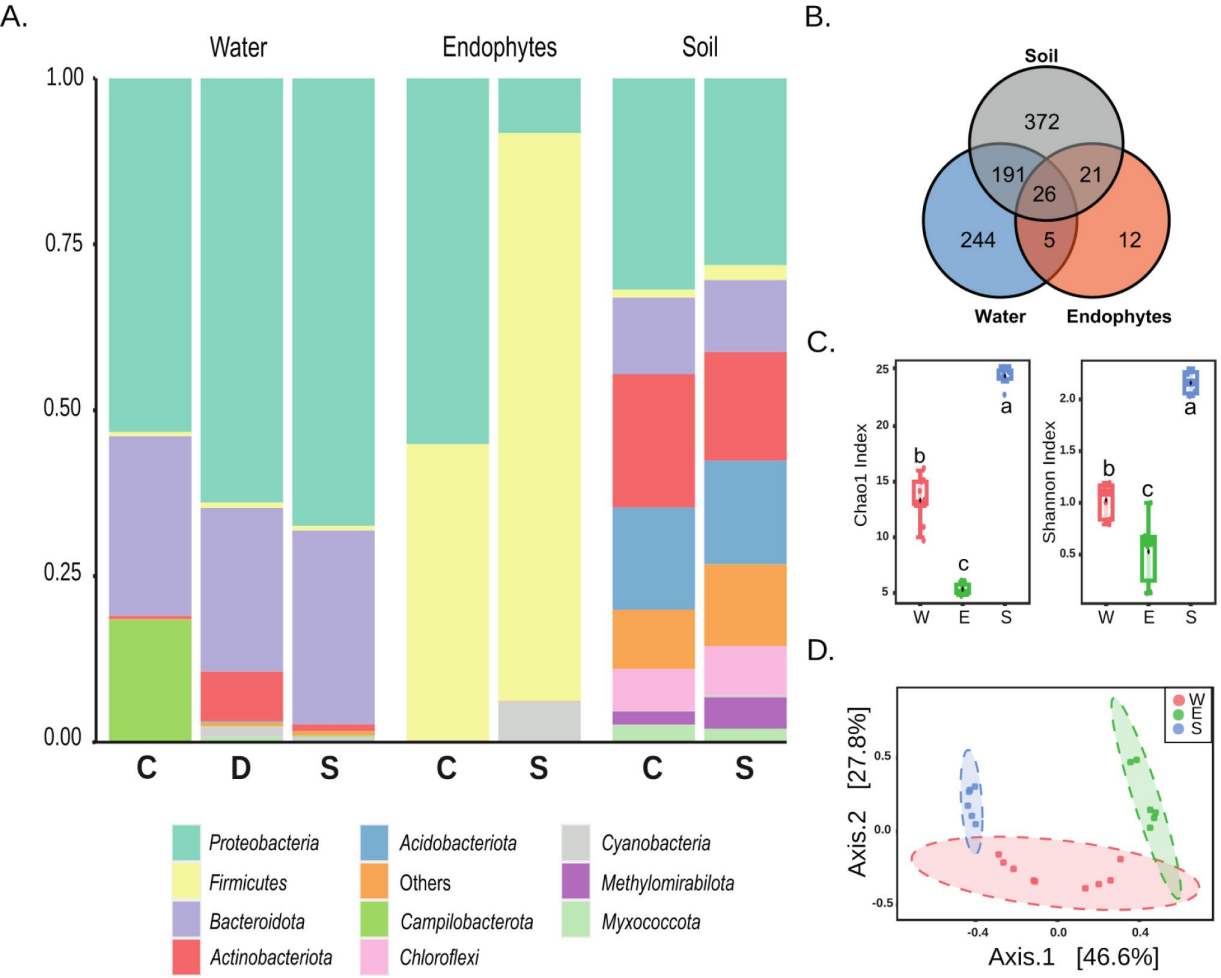
Bacterial diversity in samples from sugarcane plant endophytes, sugarcane rhizospheric soil, and irrigation water. **A** Relative abundances at the phylum level for Carmelita (C), Santa Monica (S), and District (D). **B** Venn diagram at the genus level, grouping samples from the different locations for the three matrices. **C** Alpha diversity indices (Chao1 and Shannon). **D** Principal component analysis (PCA) for the three matrices

Water, rhizospheric soil, and plant tissue (sugarcane internodes) samples were collected during the dry season (July and August 2024) from two sugarcane farms: Carmelita (Roldanillo municipality, upstream, 4°42′634″N 76°13′633″W) and Santa Mónica (Toro municipality, downstream, 4° 59′ 700″ N 76° 05′ 083″ W), located in the middle basin of the Cauca River within the ASORUT irrigation district. From each farm, four composite irrigation water samples (15.2 L each) were collected. The water was filtered to remove solid residues larger than 8 µm (BOECO qualitative filters) prior to DNA extraction. For each farm, three composite soil/root samples were prepared by combining 15 subsamples of sugarcane roots and associated soil extracted from a depth of 0–20 cm. These subsamples were obtained along transects spanning approximately 25 m across the plot. Additionally, three composite soil samples were collected from each farm for physicochemical analysis (Fig. 1S). The methodology and detailed results of the soil physicochemical analyses are provided in the Supplementary Material (SM). Sixteen whole sugarcane stalks were randomly selected from four representative quadrants at each farm. The stalks were cut 30 cm above the first internode and sectioned to generate three composite samples per farm. Sugarcane segments were wrapped in sterile absorbent towels and then sealed in sterile bags. All collected samples were stored at 4 °C and processed within 24 h of collection.

### DNA extraction and sequencing

DNA was extracted in triplicate from all environmental samples (rhizospheric soil, irrigation water, and sugarcane tissues) using the DNeasy® PowerSoil Pro® Kit (Qiagen, Hilden, Germany), following the manufacturer’s protocol with specific modifications for irrigation water and plant tissue samples. For irrigation water samples, 2 L of each sample were filtered through 0.22 μm polyamide membranes (GE Healthcare Life Sciences Whatman™). The membranes were aseptically sectioned with sterile instruments and transferred to the PowerBead Pro lysis tubes from the extraction kit. Plant tissues were surface-sterilized by immersion in a 2% sodium hypochlorite solution containing 0.1% Tween 20 for 3 min (D’Amico et al. 2008). Five grams of disinfected plant tissue were pulverized in liquid nitrogen to obtain a homogeneous powder. PCR amplification was performed using 30 ng of high-quality template DNA and primers specific for the V3-V4 hypervariable region of the 16S rRNA gene (338F and 806R). PCR products were purified with Agencourt AMPure XP magnetic beads (Beckman Coulter™), eluted in TE buffer, and tagged with adapters to complete library construction. Library quality was assessed using an Agilent 2100 Bioanalyzer (Agilent Technologies, Santa Clara, CA, USA). Only libraries with insert sizes between 400 and 450 bp and concentrations exceeding 2 nM were selected for sequencing on the Illumina™ HiSeq 2500 platform, following standard manufacturer protocols.

### Bioinformatic

Raw 16S rRNA sequencing data for the water, soil, and sugarcane endophyte samples are available in the NCBI Sequence Read Archive (SRA) under Bioproject accession number PRJNA1439857. Paired-end sequences for the V3–V4 region of the 16S rRNA gene were assembled using PEAR v.0.9.6 (Zhang et al. 2014) with default parameters. After primer removal, sequences were quality-filtered based on criteria including a quality score (Q = 30), a minimum expected error (0.1), and adequate length (> 400 bp). Chimeric sequences were removed using VSEARCH v.2.15.1 (Rognes et al. 2016) with the SILVA v.138 reference method. Sequence dereplication was performed with VSEARCH. Sequences were clustered at 100% similarity to generate zero-radius operational taxonomic units (zOTUs) using USEARCH v.11 (Edgar 2010). Taxonomic classification of sequences was performed with the SINTAX command (Edgar 2016) using the SILVA v.138 database and an 80% confidence threshold. Functional predictions were categorized according to the KEGG database, providing a detailed characterization of the metabolic pathways present in the studied microbial communities.

#### Analysis of xenobiotics in water and endophyte matrices

PhACs were determined using an ultra-high-performance liquid chromatography system (Waters, Milford, MA, USA) coupled to a Xevo TQS triple quadrupole mass spectrometer (Waters). Detailed information regarding the instrumentation, chromatographic separation, and mass spectrometry conditions is provided in the SM (LC–MS/MS analysis). The analytical method focused on the quantification of ten PhACs: valsartan, diclofenac, clarithromycin, carbamazepine, ciprofloxacin, sulfamethoxazole, enalapril, metronidazole, trimethoprim, and venlafaxine. Irrigation water samples were processed as follows: 50 mL aliquots of three-day composite samples were placed in Falcon tubes and transported to the laboratory at 4 °C within 24 h of sampling. Samples were stored at −20 °C until analysis. On the day of analysis, samples were thawed at room temperature, transferred to 2 mL Eppendorf tubes, and centrifuged at 12,000 rpm for 3 min. The multi-residue LC–MS/MS method used for water analysis was based on our previous work and involved direct sample injection without pre-concentration, with the addition of the corresponding isotopically labeled internal standards (ILIS) to correct for matrix effects correction (Moncayo-Lasso et al. 2025). The procedure involved taking 950 μL of centrifuged water samples and adding 50 μL of ILIS mix at 20 μg L^−1^ (final concentration of ILIS in injected samples: 1 μg L^−1^). Finally, 50 μL were directly injected into the LC–MS/MS system. The lowest calibration level (5 ng L^−1^) was established as the limit of quantification (LOQ) for the compounds.

Sugarcane samples were stored at ≤  − 18 °C until analysis. The LC–MS/MS method for PhACs determination in sugarcane was validated in accordance with the SANTE/11312/2021 v2 guidelines. Prior to analysis, frozen sugarcane leaves were triturated with dry ice using a Blixer 8 V.V. homogenizer. Approximately 5 g of a representative sample was weighed into a 50 mL polypropylene screw-cap centrifuge tube. Subsequently, 50 µL of ILIS solution (2.203 ng µL⁻^1^) and 10 mL of acetonitrile were added, and the sample was manually shaken for 1 min. A QuEChERS extraction kit containing 4 g magnesium sulfate, 1 g sodium chloride, 0.5 g sodium hydrogencitrate sesquihydrate, and 1 g sodium citrate was then added. The tube was shaken in a rotary agitator for 30 min and centrifuged at 4500 rpm for 5 min. After centrifugation, 0.1 mL of raw extract was diluted to 1 mL with 0.9 mL of leaf blank extract, obtained from sugarcane free of target PhACs and prepared following the same extraction procedure to account for matrix effects. The tenfold diluted extract was transferred to an injection vial, and 50 µL was injected into the LC–MS/MS system.

To ensure the reliability of the analytical results, quality control (QC) samples were prepared and analyzed alongside both water and sugarcane samples. For irrigation water, QC samples were prepared using real samples from the study, which were spiked with the target PhACs at three concentration levels (0.01, 0.1, and 1.0 μg L^−1^). QC preparation involved adding 50 μL of a mixed standard solution at concentrations of 0.2, 2.0, or 20 μg L^−1^ and 50 μL at 20 μg L^−1^ of an isotopically labeled internal standard (ILIS) mixture to 900 μL of the water sample. Quality control samples for sugarcane were prepared by fortifying blank sugarcane samples with a mixed standard solution at concentration levels of 0.01 and 0.1 mg kg^−1^. Fortification was performed by adding 50 μL of the mixed standard solution at 1 μg mL^−1^ or 10 μg mL^−1^ to 5 g of blank sample to obtain final spiking levels of 0.01 mg kg^−1^ and 0.1 mg kg^−1^, respectively, along with 50 μL of an ILIS mixture at 2 μg mL^−1^. The QC results were considered satisfactory, with recovery values ranging from 60 to 140%, and predominantly between 70 and 120%, which are within the acceptable limits established by international guidelines for residue analysis (SANTE/11312/2021 v2 2021).

Analyte quantification was performed using solvent-based calibration for water samples and matrix-matched calibration for sugarcane samples. Quantification was based on the relative peak area ratios of analytes to isotopically labeled internal standards (ILIS) using the quantifier transition (Q), allowing correction of matrix effects. Compound identification was achieved by comparing ion ratios between qualifier (q1, q2) and quantifier (Q) transitions and retention times with those of reference standards. Positive identification was confirmed when ion ratios and retention times were within the tolerance limits established by SANTE/11312/2021 v2 guidelines (± 30% for ion ratio and ± 0.1 min for retention time, respectively) (Moncayo-Lasso et al. 2025).

### Statistical analysis

Statistical analyses and data visualizations were primarily performed using the MicrobiomeAnalyst 2.0 platform and TBtools (v.2.210). Raw taxonomic counts were filtered to remove low-quality features (minimum count: 4; prevalence: 10%; low variance filter: 10% based on interquartile range, IQR). Data were normalized using Cumulative Sum Scaling (CSS) and further transformed using a Log2 scale to stabilize variance and handle the high dynamic range of microbial abundances. Alpha diversity (Chao1 and Shannon indices) was compared using the non-parametric Kruskal–Wallis test for multi-group comparisons and the Wilcoxon rank-sum test for pairwise comparisons (e.g., between Carmelita and Santa Mónica sites), with significance set at *p* < 0.05. Beta diversity was assessed through principal component analysis (PCA) based on the Bray–Curtis distance at the feature level, with community structure differences statistically validated by PERMANOVA (999 permutations). Venn diagrams were constructed to identify and visualize shared and unique genera across the water-soil-plant continuum. High-resolution heatmaps for the most abundant and pathogenic genera were generated using TBtools, applying row-scale normalization where appropriate. Functional potential was predicted via PICRUSt using the Greengenes database (v13.5). Finally, Spearman rank correlations (*p* < 0.05, rho > 0.3) were used to explore significant associations between microbial genera and PhACs concentrations, while chemical data were expressed as means ± standard error.

## Results

The physicochemical properties of the rhizospheric soil samples were analyzed to characterize the environmental conditions of the sampling sites. A detailed description of the methodology and results of these analyses is provided in the SM.

### Bacterial diversity across matrices

Bacterial diversity showed a clear filtering gradient along the continuum, decreasing sharply from water and soil to the endophytic tissues (Fig. 1C). The endophyte matrix exhibited the lowest levels of alpha diversity (Fig. 1C) and was dominated by the phyla Firmicutes (70.2%) and Proteobacteria (25.8%). In contrast, the soil matrix displayed the highest alpha diversity, with a notable presence of Proteobacteria (29.7%), Actinobacteriota (16.7%), and Acidobacteriota (16.3%) (Fig. 1A). The irrigation water matrix was predominated by Proteobacteria (59.9%), Bacteroidota (26.9%), and Campylobacterota (6.4%). Despite the differences in alpha diversity, 26 genera were shared across the three matrices (Fig. 1B). The principal component analysis (PCA) clearly separated the samples from each matrix, explaining 74.4% of the total variability (*R*^2^ = 0.80, *p* < 0.001) (Fig. 1D).

### Bacterial diversity in irrigation water

**Fig. 2:**
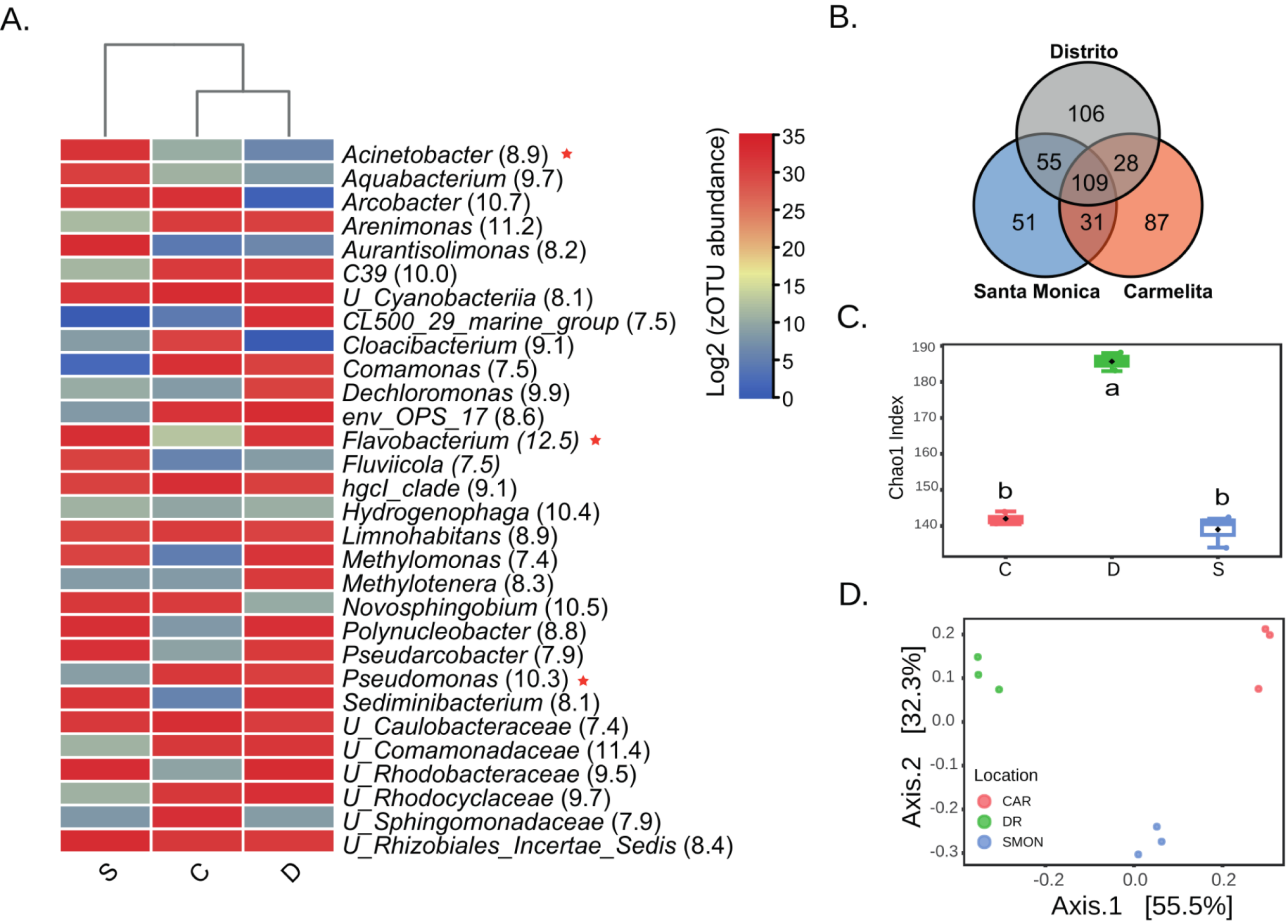
Bacterial diversity in irrigation water samples from three municipalities. **A** Relative abundance at the genus level for Santa Monica, Carmelita, and District at two sampling times. Genera marked with a star are associated with pathogenic potential. **B** Venn diagram at the genus level for Santa Monica vs. Carmelita vs. District. **C** Chao1 diversity indices. **D** PCA for all municipalities

A total of 466 bacterial genera were detected. The most prevalent genus was *Flavobacterium* (19.8%), followed by *Arenimonas* (8.4%), *Arcobacter* (5.9%), *Novosphingobium* (5.1%), and *Hydrogenophaga* (4.4%) (Fig. 2A). Of the 466 genera, 109 (23.4%) were shared among the District, Santa Mónica, and Carmelita, while 55 (11.8%) and 28 (6.0%) genera were shared exclusively between the District–Santa Mónica and District–Carmelita pairs, respectively. Furthermore, exclusive genera were identified for each location: District (106), Santa Mónica (51), and Carmelita (87) (Fig. 2B). Alpha diversity analysis using the Shannon index revealed that the District exhibited the highest diversity with significant differences compared to the other sampling sites (*p* < 0.05, Kruskal–Wallis test), whereas Carmelita and Santa Mónica showed similar indices (Fig. 2C). The PCA demonstrated a clear separation of samples by location, explaining 87.8% of the total variability (*R*^2^ = 0.83, *p* = 0.009) (Fig. 2D).

### Bacterial diversity in sugarcane rhizosphere

**Fig. 3:**
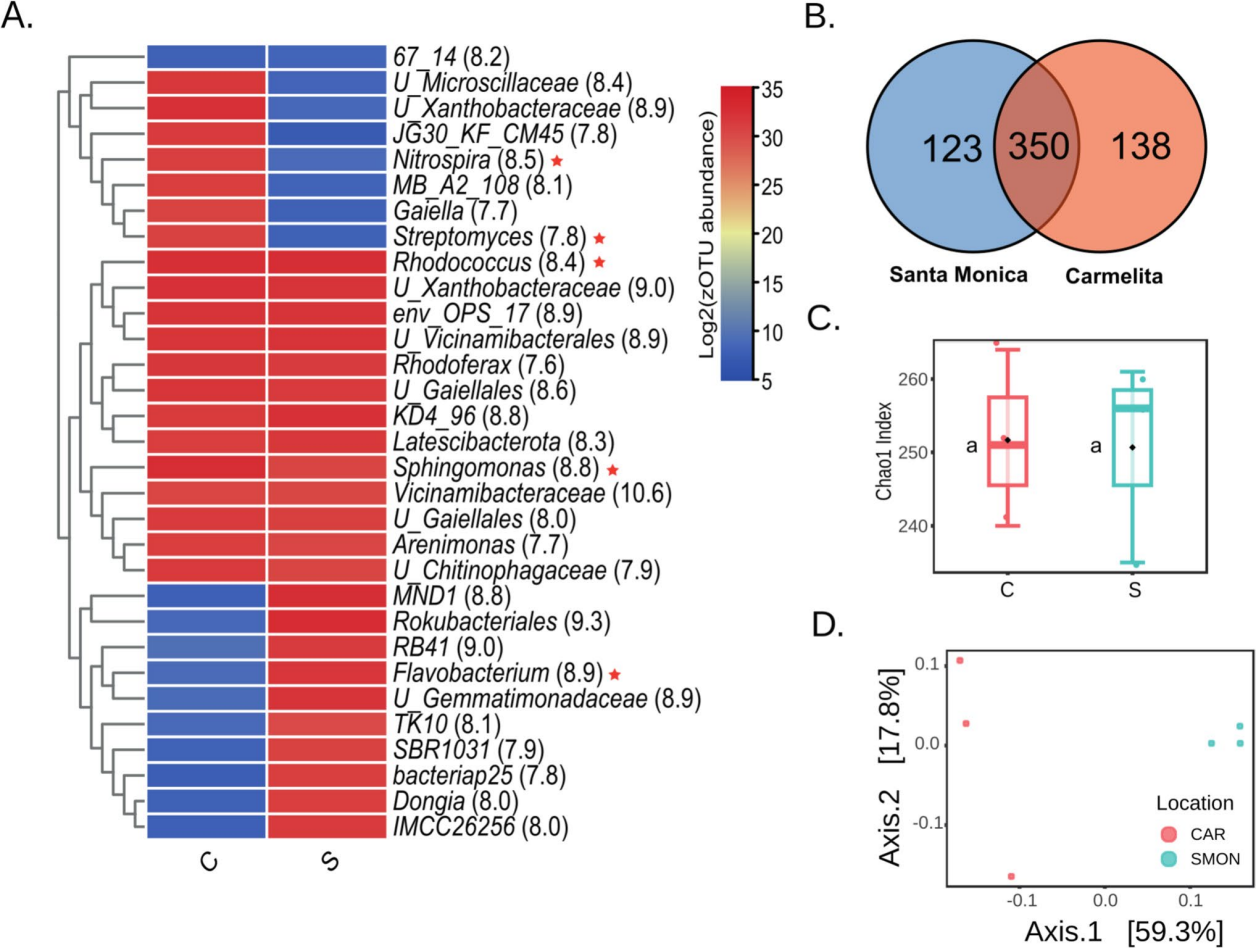
Bacterial diversity in sugarcane rhizosphere samples from two municipalities. **A** Relative abundance at the genus level for Carmelita (C) and Santa Mónica (S). Genera marked with a star are associated with pathogenic potential. **B** Venn diagram at the genus level for Santa Monica vs. Carmelita. **C** Chao1 diversity indices. **D** PCA for Carmelita (C) and Santa Mónica (S)

A total of 611 bacterial genera were detected. The most prevalent genus was Unclassified_Vicinamibacteraceae (7.8%), followed by *Rokubacteriales* (3.0%), Unclassified_Xanthobacteraceae (2.6%), and *Flavobacterium* (2.3%) (Fig. 3A). Of the 611 genera, 350 (57.3%) were shared between the Carmelita and Santa Mónica samples, while 123 (20.1%) and 138 (22.6%) genera were exclusive to Santa Mónica and Carmelita, respectively (Fig. 3B). Alpha diversity analysis using the Shannon index revealed no significant differences between the Carmelita and Santa Mónica municipalities (*p* > 0.05) (Fig. 3C). The PCA showed a partial separation of samples by municipality, explaining 77.1% of the total variability (*R*^2^ = 0.55, *p* = 0.1) (Fig. 3D).

### Bacterial diversity in sugarcane endophytes

**Fig. 4:**
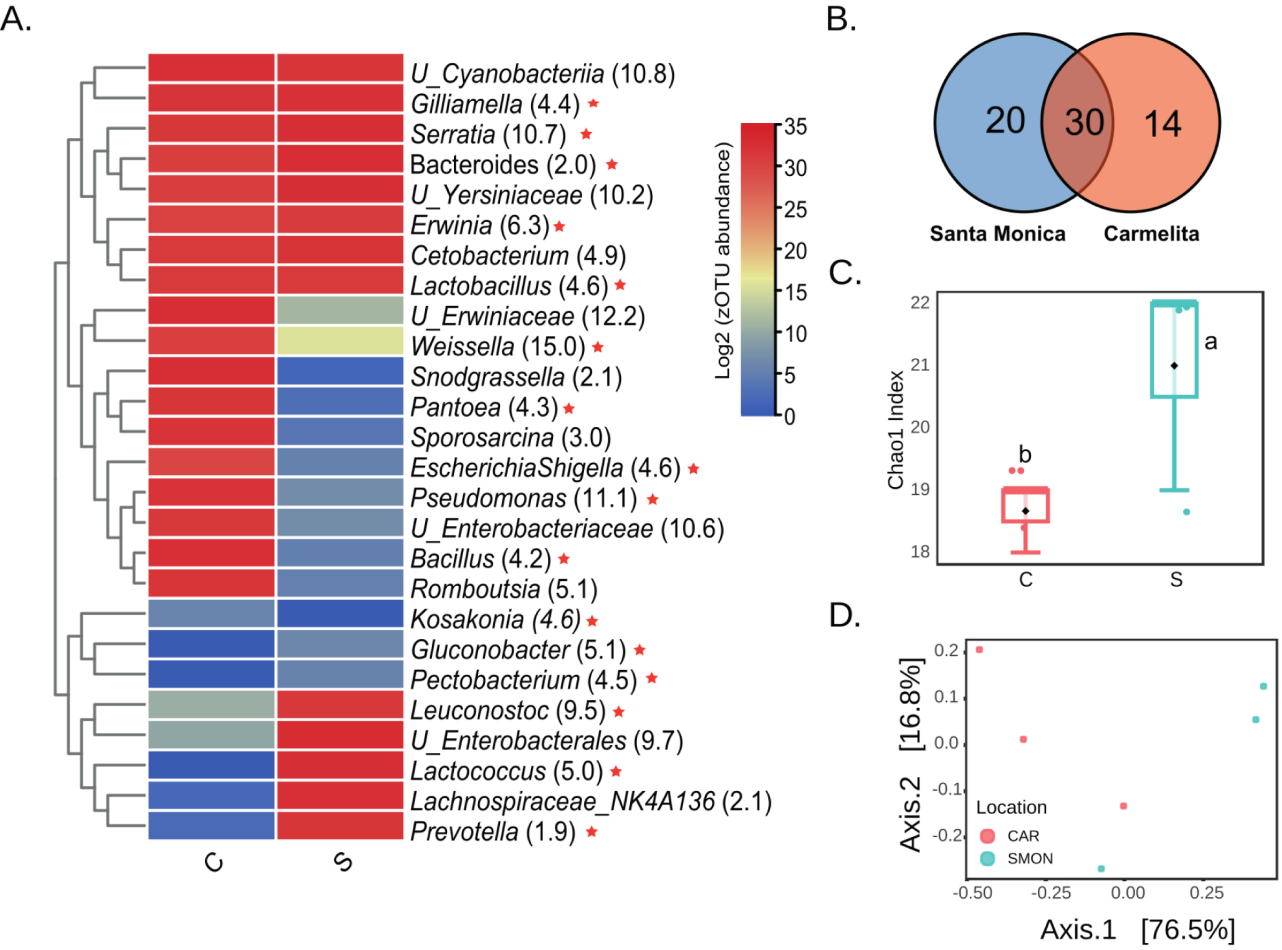
Bacterial diversity in sugarcane endophyte samples from two municipalities. **A** Relative abundance at the genus level for Carmelita (C) and Santa Mónica (S). Genera marked with a star are associated with pathogenic potential. **B** Venn diagram at the genus level for Santa Monica vs. Carmelita. **C** Chao1 diversity indices. **D** PCA for Carmelita (C) and Santa Mónica (S)

A total of 64 bacterial genera were detected. The most prevalent genus was *Weissella* (68.7%), followed by Unclassified_Erwiniaceae (9.8%), *Pseudomonas* (4.6%), Unclassified_Cyanobacteriia (3.9%), and *Serratia* (3.6%) (Fig. 4A). Of the 64 genera, 30 (46.9%) were present in both municipalities (Santa Mónica and Carmelita), while 20 (31.3%) and 14 (21.9%) genera were specific to Santa Mónica and Carmelita, respectively (Fig. 4B). Alpha diversity analysis using the Shannon index showed significant differences between the Carmelita and Santa Mónica municipalities (*p* < 0.05, Wilcoxon test) (Fig. 4C). The PCA displayed a clear separation of samples by municipality, explaining 93.3% of the total variability (*R*^2^ = 0.53, *p* = 0.2) (Fig. 4D).

### Detection of bacteria with pathogenic potential

**Fig. 5:**
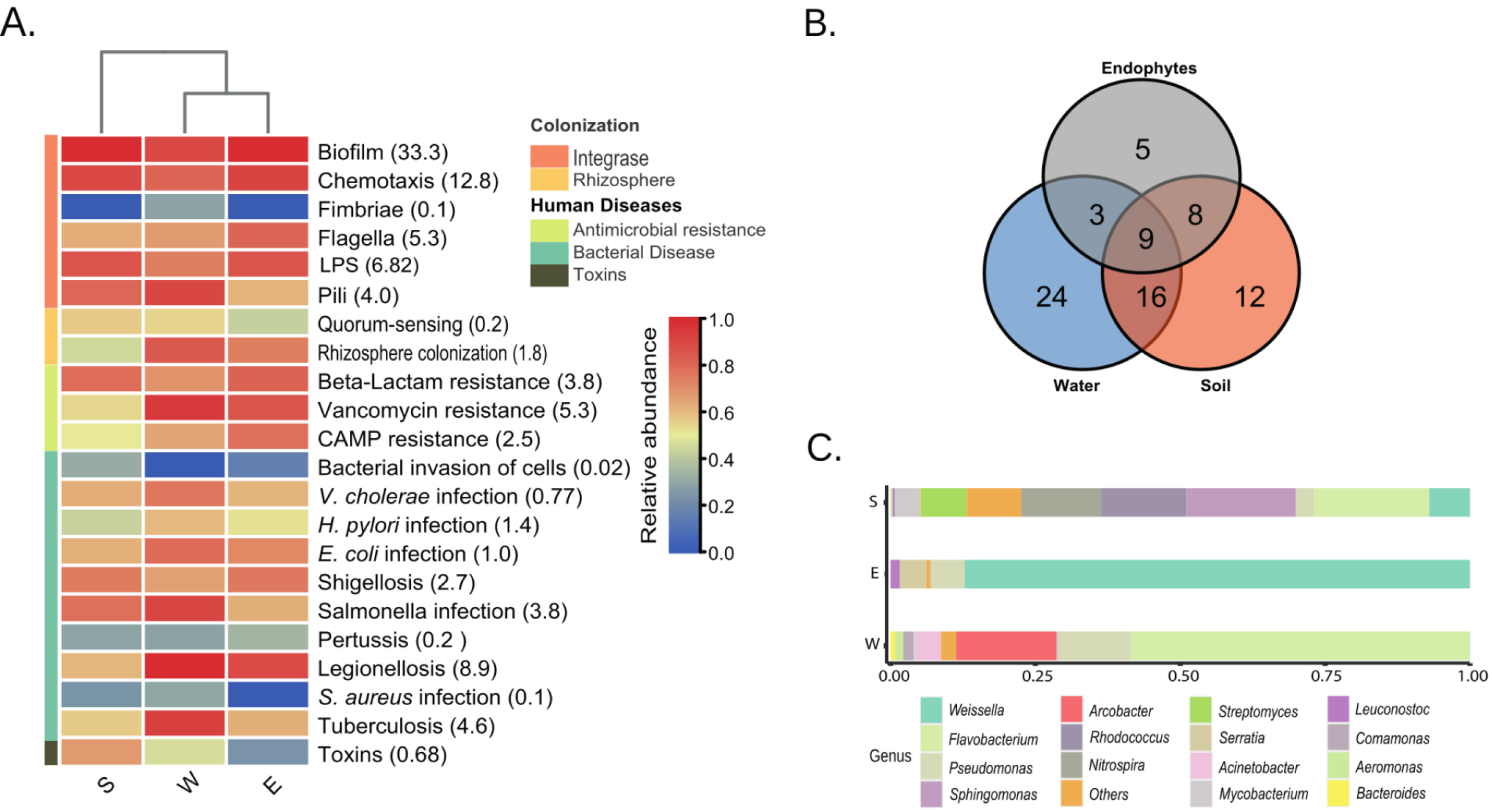
Analysis of pathogenic bacterial diversity in samples from sugarcane plant endophytes, sugarcane rhizospheric soil, and irrigation water. **A** Exploration of mechanisms linked to KOs found for potentially pathogenic genera. **B** Representation of relative abundances at the genus level in soil (S), water (W), and endophytes (E). **C** Venn diagram comparing the number of genera present in the three matrices. **D** Graphical representation of the correlation between genera in soil (blue), endophytes (orange), and water (green); blue bars represent negative relationships, while red bars represent positive relationships

The relative abundance of genera with pathogenic potential varied significantly among the studied matrices (*p* < 0.05, Kruskal–Wallis test). In the endophyte matrix, the most prevalent genera were *Weissella* (87.2%), *Pseudomonas* (5.9%), *Serratia* (4.6%), and *Leuconostoc* (1.6%). In the water matrix, *Flavobacterium* (58.5%), *Arcobacter* (17.3%), *Pseudomonas* (12.7%), *Acinetobacter* (4.4%), and *Comamonas* (1.8%) were prominent. Finally, in the soil matrix, the most representative genera were *Flavobacterium* (19.8%), *Sphingomonas* (18.9%), *Rhodococcus* (14.8%), *Nitrospira* (13.8%), *Streptomyces* (7.9%), *Weissella* (7.0%), *Mycobacterium* (4.0%), *Pseudomonas* (3.2%), *Bacillus* (2.3%), and *Lactococcus* (1.2%) (Fig. 5B).

Genera with pathogenic potential shared across all three matrices were identified, including *Weissella*, *Pseudomonas*, *Bacillus*, *Aeromonas*, *Bacteroides*, *Lactobacillus*, *Serratia*, *Sphingomonas*, and *Escherichia-Shigella*. Additionally, genera were specifically shared between pairs of matrices. The endophyte and irrigation water matrices shared *Prevotella*, *Shewanella*, and *Erwinia*, whereas the endophyte and soil matrices shared *Rhodococcus*, *Leuconostoc*, *Pectobacterium*, *Lactococcus*, *Edwardsiella*, *Enterococcus*, *Gilliamella*, and *Streptomyces*. Notably, the water and soil matrices had the highest number of shared potentially pathogenic genera (16), including *Coxiella*, *Flavobacterium*, *Allorhizobium*, *Neorhizobium*, *Pararhizobium*, *Rhizobium*, *Treponema*, *Halomonas*, *Acinetobacter*, *Microbacterium*, *Laribacter*, *Mycobacterium*, *Brevundimonas*, *Roseomonas*, *Bifidobacterium*, *Geobacter*, *Klebsiella*, and *Stenotrophomonas* (Fig. 5C). It is important to note that genera shared between matrices do not necessarily correspond to the same zOTUs.

A total of 386 zOTUs with pathogenic potential were identified, representing 8.6% of the total zOTUs detected in the study. Analysis of KEGG Orthologs (KO) associated with these zOTUs revealed their involvement in various pathogenicity-related processes, including biofilm formation (33.3%), chemotaxis (12.8%), Legionellosis (8.9%), lipopolysaccharide biosynthesis (8.8%), flagellar assembly (5.3%), vancomycin resistance (5.3%), and pilus formation (4.0%) (Fig. 5A).

### Bacterial diversity with plant growth promotion and biocontrol potential

**Fig. 6:**
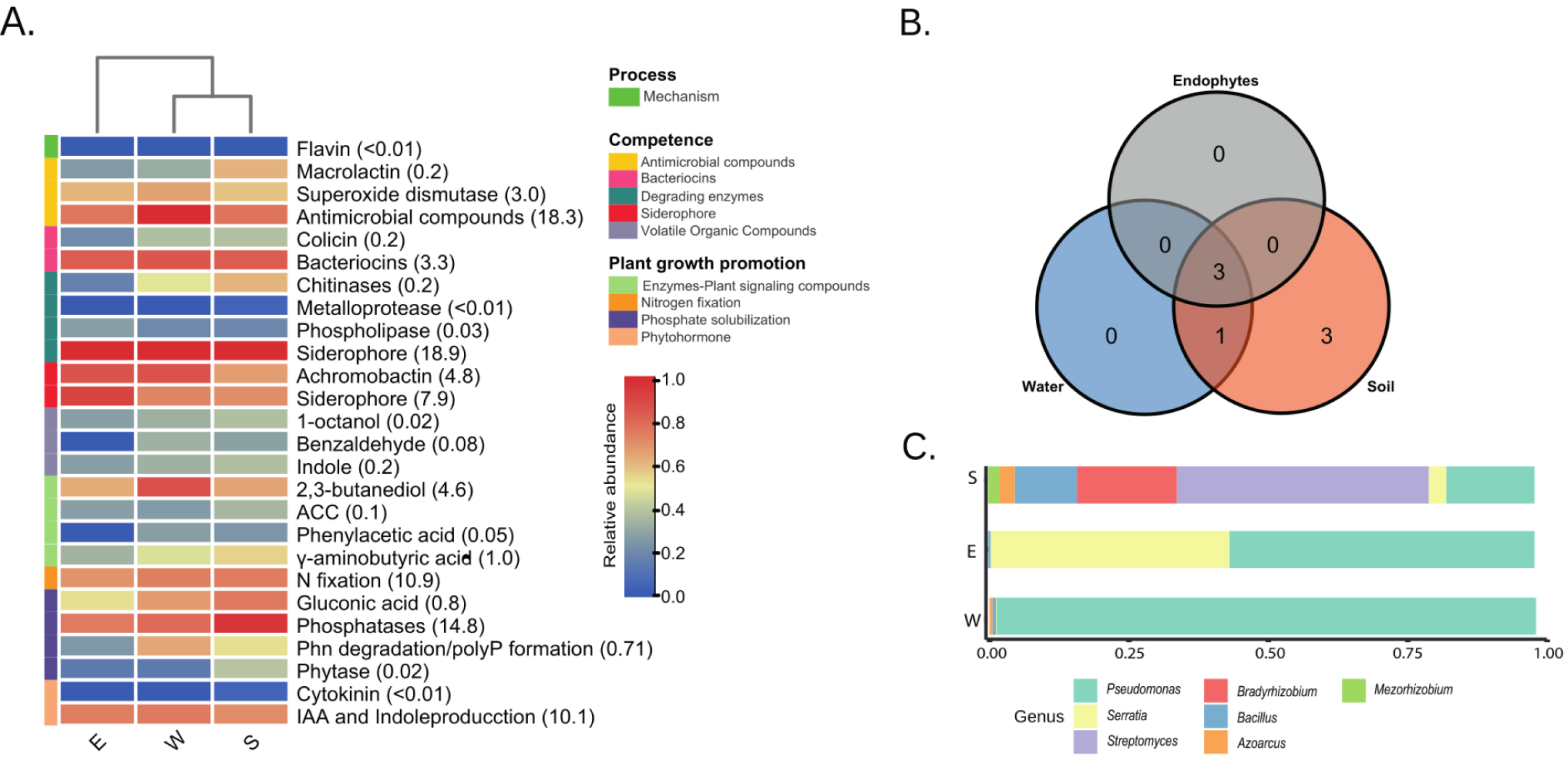
Analysis of pathogenic bacterial diversity in samples from sugarcane plant endophytes, sugarcane rhizospheric soil, and irrigation water. **A** Exploration of mechanisms linked to KOs found for genera with biocontrol, growth promotion, and nitrogen fixation potential. **B** Representation of relative abundances at the genus level in soil (S), water (W), and endophytes (E). **C** Venn diagram comparing the number of genera present in the three matrices

Among the genera present in all three matrices, *Pseudomonas* (56.9%), *Azoarcus* (1.1%), and *Bacillus* (4.15%) are notable for their plant growth-promoting potential. For biocontrol, genera such as *Pseudomonas*, *Bacillus*, *Streptomyces* (15.6%), and *Serratia* (15.7%) were identified. Regarding nitrogen fixation, the genera *Pseudomonas*, *Azoarcus*, *Bradyrhizobium* (6.1%), and *Mesorhizobium* (0.7%) were prominent (Fig. 6B). The relative abundance of genera with biotechnological potential varied significantly across the matrices (*p* < 0.05). Genera shared among the three matrices were *Bacillus*, *Pseudomonas*, and *Serratia*. The water and soil matrices shared the genus *Azoarcus*, while the soil matrix presented three exclusive genera: *Bradyrhizobium*, *Mesorhizobium*, and *Streptomyces* (Fig. 6C). It is important to note that genera shared between matrices do not necessarily correspond to the same zOTUs. The analysis of KOs associated with zOTUs having biotechnological potential revealed various mechanisms related to plant growth promotion, biocontrol, and nitrogen fixation (Fig. 6A). The most prevalent mechanisms include the production of antimicrobial compounds (21.4%), bacteriocins (3.5%), degradative enzymes (19.0%), siderophore production (12.7%), plant-signaling compounds (5.7%), nitrogen fixation (10.9%), phosphate solubilization (16.3%), and phytohormones (10.1%).

### Correlation analysis

**Fig. 7:**
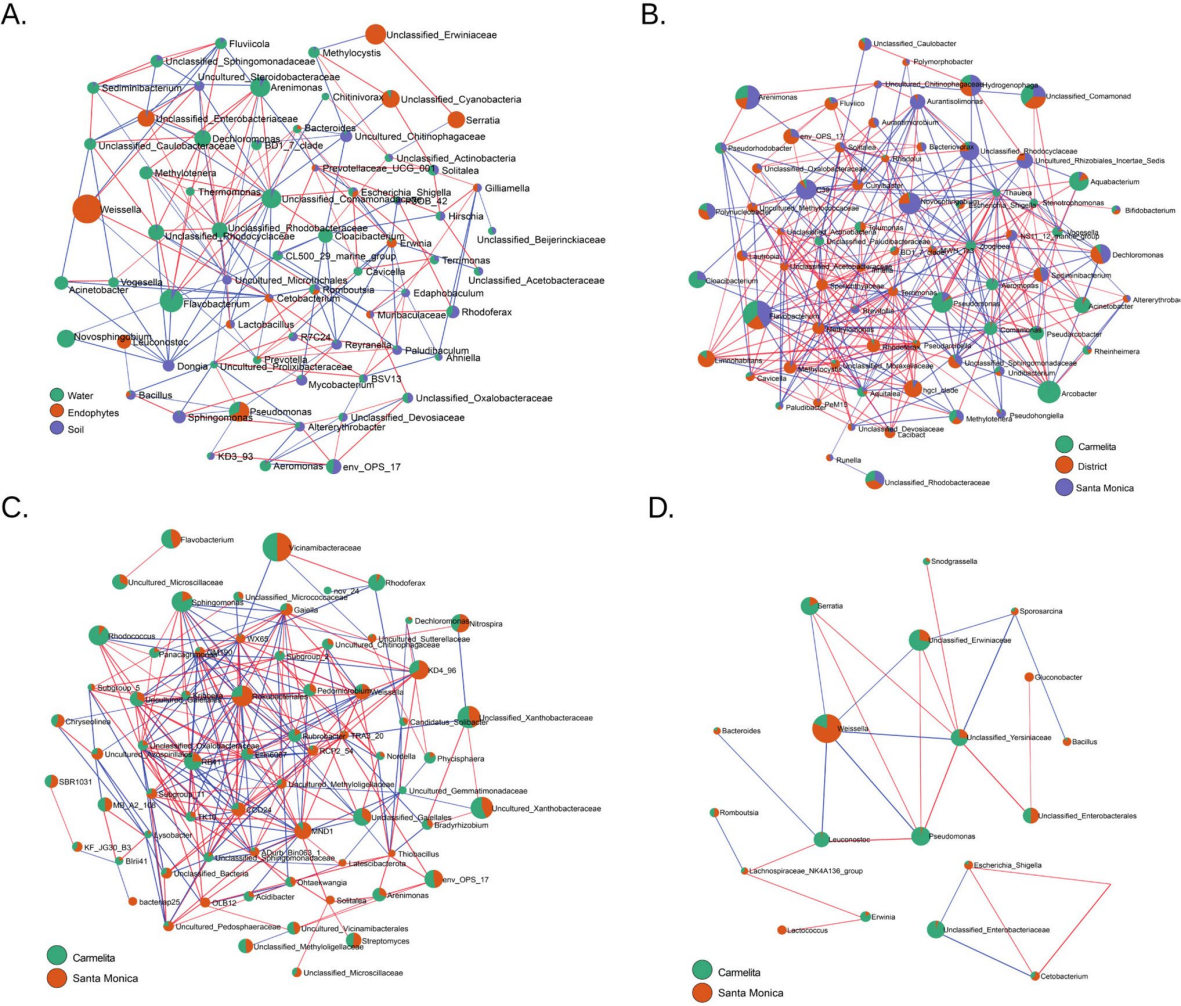
Correlation analysis of bacterial diversity in samples from sugarcane plant endophytes, sugarcane rhizospheric soil, and irrigation water. **A** Correlation analysis of the most abundant genera for the soil, water, and endophyte matrices. **B** Correlation analysis of the most abundant genera for the water matrix in Santa Mónica, Carmelita, and the District. **C** Correlation analysis of the most abundant genera for the soil matrix in Santa Mónica and Carmelita. **D** Correlation analysis of the most abundant genera for the endophyte matrix in Santa Mónica and Carmelita

The microbial correlation network revealed a complex interaction among bacterial genera in the water, soil, and endophyte matrices (Fig. 7). Both positive (red lines) and negative (blue lines) associations were observed, indicating potential co-occurrence or competitive exclusion relationships between taxa. Nodes corresponding to genera characteristic of the endophyte matrix, such as *Weissella*, *Pseudomonas*, *Serratia*, and Unclassified_Erwiniaceae, were larger and had a considerable number of connections, suggesting a hub role in the microbial network (Fig. 7). On the other hand, soil genera like *Sphingomonas* and *Rhodoferax* showed multiple correlations, both positive and negative, with bacteria from the other matrices, suggesting potential microbial exchange mechanisms between environments. Similarly, several genera associated with the water matrix, such as *Flavobacterium*, *Acinetobacter*, and *Novosphingobium*, displayed a high density of interactions with bacteria from other matrices, suggesting their importance in microbiome connectivity (Fig. 7). These observations suggest an interconnected microbial network across matrices, where certain genera may act as key ecological bridges, facilitating functional exchange between niches.

### Analysis of xenobiotics in water and endophyte matrices

The results obtained for PhACs concentrations in water and plant tissue samples from the Santa Mónica and La Carmelita municipalities show several trends (Table 1). In water samples, the highest values corresponded to Valsartan, with concentrations of 734 ng L^−1^ in Santa Mónica and 1309 ng L^−1^ in La Carmelita, suggesting a higher prevalence of this compound in La Carmelita. Regarding other compounds, Diclofenac showed significant levels in both locations, at 198 ng L^−1^ in Santa Mónica and 969 ng L^−1^ in La Carmelita. Conversely, in the plant tissue samples, most compounds were not detected. Those that were detected, such as Metronidazole and Trimethoprim, were found at low or non-quantifiable concentrations, denoted by “d,” in both locations. This could indicate a residual presence of these compounds in the plants. It is important to note that, in general, the concentrations of PhACs were higher in the water samples compared to the plant tissues, highlighting the potential for these contaminants to transfer from water to plants.

**Table 1:** Concentrations of detected PhACs in water and sugarcane tissue samples from the Santa Mónica and La Carmelita locations

	Water (ng L^−1^)		Plant sample (ng g^−1^)	
Compounds	Santa Monica	La Carmelita	Santa Monica	La Carmelita
Valsartan	734.0	1309.0	-	-
Diclofenac	198.0	969.0	d	d
Clarithromycin	101.0	169.0	d	-
Carbamazepine	141.0	69.0	d	d
Ciprofloxacin	80.0	104.0	-	d
Sulfamethoxazole	49.0	126.0	d	d
Enalapril	47.0	64.0	-	-
Metronidazole	43.0	53.0	-	-
Trimetroprim	37.0	33.0	d	d
Venlafaxina	d	5.0	-	-

The symbol “d” indicates that the compound was detected concentration < LOQ (< 5 ng L^−1^ or ng g^−1^) but could not be quantified, while “-” indicates that the compound was not detected in the sample

## Discussion

This study reveals a distinct selection gradient across the water-soil-plant continuum in sugarcane crops, where PhACs in irrigation water do not translate into significant accumulation in plant tissues. Instead, this environmental pressure appears to shape a resident microbiome that, while harboring risks from the presence of opportunistic pathogens, also exhibits remarkable biotechnological potential for growth promotion and biocontrol.

### Bacterial biodiversity in irrigation water: a reservoir of risk and opportunity

The irrigation water from the Cauca River emerged as a complex microbial reservoir, introducing high taxonomic (466 genera) and functional richness into the agroecosystem. The prominence of genera such as *Flavobacterium*, *Arenimonas*, and *Arcobacter* (Fig. 2A) is indicative of impacted aquatic ecosystems, with taxa capable of actively participating in nutrient cycling (Vigneron et al. 2021). Beyond general diversity, bioinformatic analysis predicted that this microbial community represents the primary source of biological risk to the system, with a significant abundance of genes associated with bacterial diseases like Legionellosis and Shigellosis, and with antibiotic resistance (Fig. 5A).

This potential pathogenic load is reinforced by the presence and abundance of genera known to harbor opportunistic species, such as *Flavobacterium*, *Escherichia-Shigella*, and *Pseudomonas*. The coexistence of virulence genes with the PhACs detected in the water suggests an environment of co-selection, where chemical and biological pressures intertwine to shape an adapted and potentially hazardous microbiome (Bengtsson-Palme & Larsson 2016). The identification of nine pathogenic genera shared across the entire water-soil-plant continuum (Fig. 5C) reinforces the hypothesis that water is the starting point for the internal colonization of sugarcane.

Nevertheless, this same high-pressure selective environment also enriches the water with valuable biotechnological potential. Our analysis predicted a wide array of beneficial functions, including the production of antimicrobial compounds, nitrogen fixation, and siderophore production (Fig. 6A). Notably, the genus *Pseudomonas*, which accounted for the highest abundance of zOTUs with biotechnological potential in the water, stands out as an ideal candidate for the isolation and study of plant growth-promoting strains. Therefore, irrigation water should not be viewed solely as a source of contamination, but as a double-edged reservoir that simultaneously introduces health risks and opportunities for more sustainable agriculture.

### Rhizospheric bacterial biodiversity in sugarcane crops

The sugarcane rhizosphere emerged as the epicenter of microbial diversity in this system, harboring the greatest alpha and beta richness (Fig. 2). This environment, enriched by root exudates and in direct contact with irrigation water, functions as a zone of intense interaction and selection, where a community with a marked dual potential is assembled. On one hand, the abundance of genera such as those belonging to the Vicinamibacteraceae family, which are capable of reducing sulfur and iron compounds under anaerobic conditions (Huber & Overmann 2018), suggests the presence of key functional guilds for maintaining soil resilience against stresses like flooding.

However, this same richness and connectivity make the rhizosphere a niche for opportunistic pathogens. Genera like *Sphingomonas* and *Rhodococcus*, which in our study showed a high degree of connectivity in the microbial network (Leelastwattanagul et al. 2023), contain species such as *S. paucimobilis* and *R. equi*, known for their virulence factors in animal and human hosts (Saeb et al. 2014; Salazar-Rodríguez et al. 2021). This finding underscores the critical importance of the rhizosphere not only as a reservoir of benefits for the plant but also as a potential transfer hub for health risks into the food chain.

Despite the risks, the beneficial potential of this community is supported by specific molecular mechanisms. Our bioinformatic analysis predicted the presence of 137 zOTUs carrying the *acdS* gene (K01505), whose ACC deaminase enzyme is fundamental for mitigating abiotic stress in plants (Shukla et al. 2022). Furthermore, the identification of 2835 zOTUs with the *ppa* gene (K01507), associated with inorganic pyrophosphatase, points to phosphate solubilization as a key plant growth-promoting mechanism in these soils, highlighting the valuable microbial capital housed within the rhizosphere.

### Endophytic diversity associated with sugarcane plants

The endophytic community represented the final outcome of the system’s selective filter, showing a drastic reduction in diversity and a clear dominance by the genus *Weissella* (68.7% relative abundance, Fig. 4). The prominence of this genus, known for its roles in the food industry (Teixeira et al. 2021), is supported by our finding of the K03627 gene, associated with bacteriocin MBF (Fig. 6A), suggesting an endogenous antimicrobial potential. However, *Weissella* embodies the functional duality of the sugarcane microbiome, as certain strains are recognized as opportunistic pathogens in immunocompromised hosts (Fairfax et al. 2014; Vásquez-Machado et al. 2020). This coexistence of probiotic and pathogenic potential in the dominant genus underscores the complexity of defining the role of endophytes based solely on taxonomy.

Microbial interactions within this restricted niche appear to be governed by intense competition and communication. Co-occurrence analysis revealed a negative relationship between *Weissella* and other important genera such as *Pseudomonas* and *Serratia*, which could be explained by resource competition or the production of antimicrobial compounds (Tambong et al. 2022; Al-Ghanem 2018). On the other hand, the positive relationship observed between *Pseudomonas* and *Serratia* suggests potential synergies, possibly mediated by communication systems like quorum sensing (Fig. 5A), which regulates virulence and biofilm formation (Kostylev et al. 2019).

Beyond competition, these endophytes also exhibit potential for plant growth promotion. Our analysis predicted that genera like *Pseudomonas* and *Serratia* possess the mechanisms to produce key metabolites such as indole-3-acetic acid (IAA) and 2,3-butanediol (Meliani et al. 2017; Sun et al. 2023). These findings suggest that, despite the risks, the endophytic community is not passive but actively participates in plant physiology. Understanding this complex network of interactions is fundamental to developing strategies that can modulate the endophytome, enhancing its benefits while mitigating its inherent risks.

### Xenobiotics in irrigation water and plant tissues

The PhACs profile detected in the irrigation waters of the Cauca Valley reveals a concerning pattern of environmental exposure. The antihypertensive valsartan (734–1309 ng L^−1^) and the anti-inflammatory diclofenac (198–969 ng L^−1^) predominated in all samples, with concentrations that considerably exceed ecotoxicological risk thresholds (Mezzelani et al. 2018). These levels, consistent with those reported in other Colombian watersheds impacted by urban effluents (Botero-Coy et al. 2018), confirm that the sugarcane agroecosystem is under constant chemical pressure. Critically, the simultaneous detection of antibiotics like Ciprofloxacin (80–104 ng L^−1^) at concentrations capable of selecting for resistant bacteria (Bengtsson-Palme & Larsson 2016) demonstrates that the water is not merely a vehicle for contaminants, but an active environment shaping the evolution of the microbiome that enters the rhizosphere.

However, the most striking finding of this study is the marked decoupling between this severe water contamination and the near-zero transfer of PhACs into plant tissues. This observation contrasts with numerous studies reporting measurable uptake and accumulation of PhACs in crops irrigated with reclaimed or wastewater (e.g., Goldstein et al. 2014; Malchi et al. 2014; Wu et al. 2010). These discrepancies suggest that the absorption of these compounds is highly context-dependent and likely governed by a combination of compound-specific properties, plant physiology, and analytical scope. An additional and often overlooked mechanism underlying this decoupling is soil-mediated biogeochemical filtration. During infiltration, soil organic matter and clay minerals can adsorb and immobilize xenobiotics, including PhACs and trace metals, thereby substantially reducing their bioavailability at the root interface (Mora et al. 2022). Such edaphic sequestration limits systemic translocation within the plant and may act as a primary barrier before compound-specific or physiological processes become relevant (Vázquez-Tapia et al. 2022).

From a chemical perspective, the physicochemical properties of individual compounds are critical determinants of plant uptake and translocation. Compounds such as Valsartan exhibit low aqueous solubility and limited membrane permeability, characteristics associated with restricted root uptake and xylem transport (Malchi et al. 2014). Similarly, diclofenac, although more hydrophobic, has been shown to strongly associate with root cell membranes and phospholipids, promoting retention in belowground tissues and limiting translocation to shoots (Miller et al. 2016; Prosser & Sibley 2015). In contrast, PhACs frequently reported to accumulate in plants, such as carbamazepine, tend to exhibit higher persistence and mobility within plant tissues (Fu et al. 2019).

Plant morphological and anatomical traits likely play a decisive role in this decoupling. Studies reporting substantial PhACs uptake have predominantly examined leafy vegetables or short-cycle herbaceous crops characterized by high transpiration rates and minimal structural resistance to upward contaminant transport (Goldstein et al. 2014; Fu et al. 2019; Moncayo-Lasso et al. 2025). Sugarcane, in contrast, is a perennial C4 grass with extensive lignification, dense vascular bundles, and a fibrous stem architecture. These structural features impose mechanical constraints on long-distance xenobiotic translocation, limiting contaminant movement toward the stem pith and aerial tissues. Together with biomass dilution effects, this anatomical filtration provides a plausible explanation for the minimal internal accumulation of PhACs despite sustained exposure through irrigation water.

In addition, plant metabolic transformation processes may play an important role. The high sucrose content characteristic of mature sugarcane stems can alter the physicochemical environment within vascular tissues. Elevated sugar concentrations have been reported to reduce membrane permeability and restrict the transport range of contaminants (Guo et al. 2023). Moreover, sucrose may enhance contaminant degradation (Kamal et al. 2023) and competitively interfere with xenobiotic movement within the vascular system (Soh et al. 2011). These sucrose-mediated effects provide a physiological layer that explains the restricted systemic distribution observed. Furthermore, plants can enzymatically transform PhACs into metabolites sequestered in vacuoles, which often remain undetected when only parent compounds are targeted (Goldstein et al. 2014).

Taken together, these findings suggest that sugarcane serves as a robust physiological barrier against PhACs translocation, contrasting with the accumulation patterns observed in more vulnerable agroecosystems. In Mexican agricultural regions irrigated with untreated wastewater, the systemic transfer of contaminants to edible tissues is well documented, particularly in staple crops like maize and beans. In these species, the bioaccumulation of compounds—such as valsartan, diclofenac, and ciprofloxacin—within the grains is primarily driven by the grain-filling stage, a physiological phase that facilitates the active partitioning of xenobiotics into reproductive structures (Hammad et al. 2018; Marques et al. 2021; Biczak et al. 2024). Conversely, sugarcane lacks a comparable grain-filling process; this fundamental distinction, alongside its lignified architecture and high internal sucrose concentrations, effectively restricts the concentrated accumulation of organic micropollutants within tissues destined for human consumption.

### Chemical and biological transfer in the water-soil-plant system

The integrated analysis of our study reveals that the sugarcane water-soil-plant continuum functions as a dichotomous filter with profound implications for risk assessment. On one hand, we demonstrated that it is a highly efficient barrier against the translocation of PhACs from water. On the other hand, our data strongly suggest a permeable pathway for biological transfer, evidenced by the presence of nine potentially pathogenic genera, including *Weissella*, *Pseudomonas*, and *Escherichia-Shigella*, shared across all three matrices. This selection gradient, which reduces diversity from over 600 genera in the rhizosphere to just 64 in the endophytome (Frank et al. 2017), culminates in the assembly of a highly specialized internal subpopulation.

The structure of this resident microbial community appears to be governed by complex interactions, shaped by the selective pressures of the environment. Correlation analysis suggests a competitive antagonism between the dominant endophyte *Weissella* and the opportunist *Pseudomonas*, likely mediated by bacteriocin production—a capability that represented 21.4% of the predicted metabolic pathways in the endophytic community. Crucially, the prediction of a high prevalence of genes for biofilm formation (33.3% of detected mechanisms) and antimicrobial resistance suggests that this microbiome is functionally “primed” to persist in a hostile environment—one contaminated by the PhACs that the plant blocks but that the microbes must confront.

Therefore, this study argues that safety assessments in these agroecosystems should be conceptually reframed. Rather than constituting a quantitative human health risk assessment, our findings identify environmental indicators of potential risk associated with the internalization of microorganisms carrying predicted virulence and antimicrobial resistance traits. While direct human exposure pathways—such as consumption of sugarcane-derived products or occupational contact with irrigation water—were not evaluated in this study, the observed microbial patterns provide early warning signals of conditions that may compromise public and agricultural health. From a One Health perspective (Pérez-Vidal et al. 2016), these results highlight the need for integrated monitoring strategies that couple chemical surveillance with microbiome characterization and future exposure-based assessments to enable robust public health recommendations.

Finally, several limitations should be considered when interpreting these findings. First, functional traits related to virulence, antimicrobial resistance, and plant growth promotion were inferred using predictive bioinformatic tools based on reference databases and were not experimentally validated. While such predictions provide valuable insights into potential functionality, they do not confirm actual gene expression or phenotypic activity under field conditions. Second, the chemical analysis focused on a selected group of PhACs compounds and did not include other relevant contaminants commonly associated with agricultural irrigation waters, such as pesticides or heavy metals, which may interact synergistically with microbial communities and influence selection pressures. Third, sampling was conducted exclusively during the dry season. Seasonal hydrological variability during wetter periods may alter both contaminant loads—potentially through dilution effects or increased agricultural runoff—and microbial dynamics. Over the years, chronic exposure to these fluctuating environmental pressures could drive progressive microbial adaptation, potentially increasing the prevalence and persistence of resistance traits within the crop-associated microbiome.

## Conclusions

This study demonstrates that the sugarcane water-soil-plant system functions as a highly differentiated selective filter. While this system effectively limits the translocation of PhACs contaminants into plant tissues, it simultaneously permits the internal colonization of a subset of bacteria originating from irrigation water, including potentially pathogenic genera. These findings redefine the risk paradigm in such agroecosystems: the primary hazard indicators identified in this study appear to arise not from direct chemical contamination of the crop, but from the establishment of an endophytic reservoir of microorganisms with pathogenic potential and antimicrobial resistance genes, whose assembly is shaped by environmental selective pressures. Concurrently, our analysis predicted significant biotechnological potential within this microbiome, opening new avenues for sustainable agriculture.

While the aforementioned limitations contextualize our findings, it is also important to note that our 16S rRNA gene-based analysis, although establishing strong correlations and the shared presence of taxa, cannot definitively confirm the transfer of specific bacterial strains from water to endophytic tissues. To definitively track this transfer, future longitudinal studies employing shotgun metagenomic approaches will be required. Nonetheless, despite these caveats, this work provides the first comprehensive characterization of this interface in Colombia’s sugarcane agroecosystems. Ultimately, it underscores the need to shift the risk paradigm: from solely monitoring chemical contaminants in the final product towards managing the internalized microbiome as a key component of public and agricultural health.

## Supplementary Information

Below is the link to the electronic supplementary material.

ESM1 (DOCX 1.74 MB)

## Data Availability

The raw 16S rRNA sequencing datasets generated and analyzed during the current study are available in the NCBI Sequence Read Archive (SRA) under BioProject accession number PRJNA 1439857. Other datasets used and/or analyzed during the current study are available from the corresponding author on reasonable request.
