# Supplementary material for: Microbial communities and pharmaceutical contaminants in the water-soil–plant continuum of sugarcane crops irrigated with contaminated waters from the Cauca River Valley, Colombia
Source: Environ Sci Pollut Res Int. 2026 Jun 25;33(20):10202–17. doi: 10.1007/s11356-026-37722-y (PMC13350160; doi:10.1007/s11356-026-37722-y)
Supplement: Supplementary file 1 — (DOCX 1.74 MB) [file 11356_2026_37722_MOESM1_ESM.docx]

**Microbial Communities and Pharmaceutical Contaminants in the Water-Soil-Plant Continuum of Sugarcane Crops Irrigated with Contaminated Waters**

Juan Ceballos-Castillo¹ · Rodrigo A. Echeverry-Gallego¹ · Diana Martínez-Pachón¹ · Alejandro Moncayo-Lasso¹ · Javier Vanegas¹*

¹ Research Group in Biological and Chemical Sciences, Faculty of Sciences, Universidad Antonio Nariño, Bogotá D.C., Colombia

**Supplementary Material**

**Materials and Methods**

**LC-MS/MS analysis**

Acquity UPLC™ H-Class liquid chromatography system (Waters Corp., Milford, MA, USA) interfaced to a triple quadrupole mass spectrometer Xevo TQ-STM equipped with an orthogonal Z-Spray electrospray ionization interface (ESI) (Waters Corp, Manchester, UK) was used for sample analysis. The UHPLC separation was performed using an Cortecs C18 analytical column (2.1 × 100 mm, 2.7 μm particle size, Waters Corp.) maintained at 40 ◦C. The mobile phases were (A) water and (B) methanol, both with 2 mM ammonium acetate and 0.1% formic acid, delivered at a flow rate of 0.4 mL min^-1^. The mobile phase gradient was: 0 min, 10% B; 6 min, 99% B; 8 min, 99%; 8.10 min, 10% B; and maintained until 10 min for column re-equilibration. Injection volume was 50 μL. ESI was operated in positive ionization mode (ESI+) using a capillary voltage of 1 kV. Nitrogen desolvation gas flow was set to 1200 L h^-1^ and cone gas to 250 L h^-1^, while source temperature was set to 150 °C, and desolvation temperature was 650 °C. Cone voltage and collision energies, using argon (99.995%, Nippon Gases) as collision gas, were optimized for each compound. Four selected reaction monitoring (SRM) transitions were acquired per compound (Q, quantification transition; q1 and q2, confirmation transitions). Dwell times were automatically selected in order to acquire 12 points/peak, with at least 14 ms per transition. The UHPLC-MS/MS parameters for the selected analytes and their corresponding ILIS are shown in S.I. The lowest calibration level was taken as the estimated limit of quantification, which by default were established at 5 ng L^-1^ for water samples and 5 ng g^-1^ for lettuce leaf samples. Data were acquired and processed using MassLynx 4.1 software and quantified with TargetLynx application (Waters Corp, Manchester, UK).

**Soil Physicochemical Analysis**

A composite sample from each of the two farms was analyzed (n=2). The following measurements were performed to evaluate soil characteristics: pH and electrical conductivity were measured using a potentiometer in a 1:1 soil-to-water mixture (w/v) (Rhoades et al., 1989). Exchangeable Ca, K, Mg, and Na contents at pH 7.0 were determined by atomic absorption spectrophotometry (Basta & Tabatabai, 1985). Exchangeable soil acidity, measured at pH < 5.5, was determined using the potentiometric method (Shamrikova et al., 2018). Organic carbon content was assessed using the Walkley-Black method (Walkley & Black, 1934). P availability was analyzed by colorimetry (Bray & Kurtz, 1945), while S content was assessed using UV-Vis spectrophotometric techniques (Tabatabai & Bremner, 1972). Microelements (Cu, Fe, Mn, and Zn) were extracted using a modified Olsen extraction solution and measured by atomic absorption spectrophotometry (Bunus et al., 1975). Boron was extracted using monobasic phosphate (azomethine-H) (Wolf, 1974). The Effective Cation Exchange Capacity was calculated according to the method of the *Sociedad Colombiana de la Ciencia del Suelo* in 1981. Soil texture was analyzed using the Bouyoucos hydrometer method (Bouyoucos, 1962). Total nitrogen content was determined using the semi-micro Kjeldahl method (Nelson & Sommers, 1980). A principal component analysis (PCA) was performed using R (version 4.3.1) to examine the variability in physicochemical parameters between sampling sites. A biplot was generated to visualize the relationships between variables and sites, using ggplot2 for graphical representation. Additionally, a Spearman correlation analysis (|r| > 0.7) was performed using R software to examine the relationship between soil physicochemical variables and the abundance of bacterial genera. The results were visualized using correlation plots generated with the corrplot package.

**Results**

**Soil Physicochemical Analysis**

The principal component analysis (PCA) showed that the first two components explain 85% of the total variance (Figure S1A). The first principal component (PC1), which explains 65% of the variance, was primarily associated with iron (Fe) and clay, while the second principal component (PC2), explaining 20%, was related to pH and calcium (Ca). The Carmelita and Santa Mónica farms exhibited notable differences in their Fe and Clay levels.

The correlation analysis revealed distinct patterns between soil physicochemical parameters and microbial communities. In the rhizosphere, *Rhodococcus* and Xanthobacteraceae showed a negative correlation with Fe, while *Sphingomonas* and *Rhodoferax* exhibited a positive correlation with pH and Ca. Among the endophytes (Figure S1B), *Weissella*, the most abundant genus, showed a negative correlation with Fe and a positive correlation with pH. Members of the Enterobacteriaceae family correlated positively with Ca and pH, while *Pseudomonas* presented a negative correlation with Mn and Na. Lactic acid bacteria, such as *Leuconostoc* and *Lactococcus*, correlated positively with Clay and K. In the water matrix, *Flavobacterium* and *Arenimonas* showed a positive correlation with Fe and pH, but a negative correlation with Ca. Conversely, *Arcobacter* exhibited a negative correlation with pH and Fe, but a positive one with Ca.


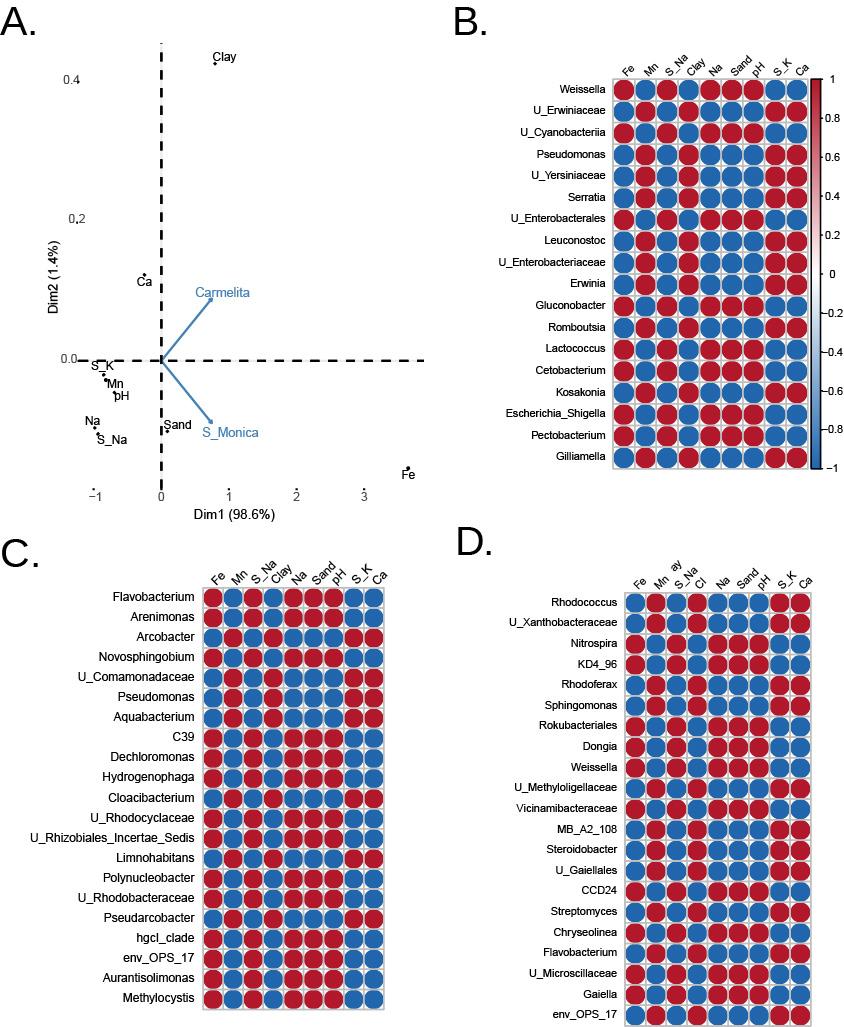


**Fig. 1S** Soil physicochemical analysis. A) Principal component analysis. B) Correlation analysis between soil physicochemical parameters and the microbial communities present in the endophyte matrix, C) Water matrix, D) Rhizosphere matrix.

**References**

Basta, N. T., & Tabatabai, M. A. (1985). Determination of exchangeable bases in soils by ion chromatography. Soil Science Society of America Journal, 49(1), 84–89. <https://doi.org/10.2136/sssaj1985.03615995004900010017x>

Bouyoucos GJ (1962) Hydrometer method improved for making particle size analyses of soils. Agron J 54(5):464-465. <https://doi.org/10.2134/agronj1962.00021962005400050028x>

Bray, R. H., & Kurtz, L. T. (1945). Determination of total, organic, and available forms of phosphorus in soils. Soil Science, 59(1), 39–46. <https://doi.org/10.1097/00010694-194501000-00006>

Bunus, F., Dumitrescu, P., & Bulaceanu, R. (1975). Analytical determination of microelements in well waters. Journal of Radioanalytical Chemistry, 27(1), 77–81. <https://doi.org/10.1007/BF02517449>

Nelson DW, Sommers LE (1980) Total nitrogen analysis of soil and plant tissues. J AOAC Int 63(4):770-778. <https://doi.org/10.1093/jaoac/63.4.770>

Rhoades JD, Manteghi NA, Shouse PJ, Alves WJ (1989) Soil electrical conductivity and soil salinity: new formulations and calibrations. Soil Sci Soc Am J 53(2):433-439. <https://doi.org/10.2136/sssaj1989.03615995005300020020x>

Shamrikova, E. V., Vanchikova, E. V., Sokolova, T. A., & otros. (2018). Potential sources of exchangeable acidity in strongly acid soils (pHKCl < 3.3) and validation of its determination. Eurasian Soil Science, 51(12), 1397–1410. <https://doi.org/10.1134/S1064229318120116>

Tabatabai, M. A., & Bremner, J. M. (1972). Distribution of total and available sulfur in selected soils and soil profiles. Agronomy Journal, 64(1), 40–44. <https://doi.org/10.2134/agronj1972.00021962006400010013x>

Walkley A, Black IA (1934) An examination of the Degtjareff method for determining soil organic matter, and a proposed modification of the chromic acid titration method. Soil Sci 37(1):29-38. <https://doi.org/10.1097/00010694-193401000-00003>

Wolf, B. (1974). Improvements in the azomethine‐H method for the determination of boron. Communications in Soil Science and Plant Analysis, 5(1), 39–44.<https://doi.org/10.1080/00103627409366478>
